# ﻿*Amphicercidus* (Hemiptera, Aphidinae, Macrosiphini) species in China: two synonyms and two new records

**DOI:** 10.3897/zookeys.1243.142124

**Published:** 2025-06-23

**Authors:** Ying Xu, Jing Chen, Bakhtiyor Rustamovich Kholmatov, Li-Yun Jiang, Ge-Xia Qiao

**Affiliations:** 1 State Key Laboratory of Animal Biodiversity Conservation and Integrated Pest Management, No. 1-5 Beichen West Road, Chaoyang District, Beijing 100101, China State Key Laboratory of Animal Biodiversity Conservation and Integrated Pest Management Beijing China; 2 College of Life Science, Ludong University, No. 186 Hongqi Middle Road, Zhifu District, Yantai City 264025, China University of Chinese Academy of Sciences Beijing China; 3 College of Life Science, University of Chinese Academy of Sciences, No. 19, Yuquan Road, Shijingshan District, Beijing 100049, China Ludong University Yantai City China; 4 Institute of Zoology, Academy of Sciences Republic of Uzbekistan, Bagishamol Str., 232b, Tashkent 100053, Uzbekistan Academy of Sciences Republic of Uzbekistan Tashkent Uzbekistan

**Keywords:** Aphids, broad cauda, DNA barcode, key, Lonicera, new record, new synonym

## Abstract

*Amphicercidus* Oestlund, 1923 is distinguished from other aphid genera by a body in life heavily covered with white wax powder, antennal segment III rather long and with numerous secondary rhinaria, body dorsal setae long and pointed, the second hind tarsal segment longer than the ultimate rostral segment, siphunculi as long stout cylinders, and cauda broad and short. Based on the examination of Chinese specimens, two species were found to be synonyms of *Amphicercidusjaponicus* (Hori, 1927): *Amphicercidusforsythiae* Zhang, Zhong & Zhang, 1992, **syn. nov.** and *Amphicercidussinilonicericola* Zhang, 1980, **syn. nov.** Additionally, *Amphicerciduspulverulens* (Gillette, 1911) and *Amphicercidustuberculatus* David, Narayanan & Rajasingh, 1971 are reported here as new records for China. Additionally, *Lonicera* (Caprifoliaceae) is a new host plant record for *Amphicerciduspulverulens*. The DNA barcodes for *A.japonicus*, *A.pulverulens* and *A.tuberculatus* have been obtained, with the barcodes of *A.pulverulens* and *A.tuberculatus* being acquired for the first time. Keys to Chinese species in this genus are presented.

## ﻿Introduction

*Amphicercidus* was erected by Oestlund (1923) who specified *Aphispulverulens* Gillette, 1911 as the type species. The main morphological characteristics of the genus are: body heavily covered with wax powder in life, antennal segment III rather long and bearing numerous secondary rhinaria, the siphunculi are stout, cylindrical and without flange (Oestlund 1923). The characters of *Amphicercidus* are similar to those of *Anuraphis*, so some species of the genus were initially described in *Anuraphis*. Hori (1927) described *Anuraphisjaponicus* feeding on *Lonicera* in Japan, Takahashi (1927) described *Anuraphislanigera* feeding on *Stauntonia* in China (Taiwan), Gillette and Palmer (1929) described *Anuraphisflocculosa* feeding on *Lonicera* in USA. They have since been transferred to *Amphicercidus* (Gillette and Palmer 1932; Hori 1938; Eastop and Hille Ris Lambers 1976). *Ferganaphisalticola* Narzikulov & Mukhamediev, 1965 was described feeding on *Lonicera* in Tajikistan, and *Ferganaphislonicericola* Blackman & Eastop, 2024 feeding on *Lonicera* was also described in Central Asia. Later, the genus *Ferganaphis* was regarded as a synonym of *Amphicercidus* (Remaudière and Remaudière 1997). Subsequently, two species feeding on *Lonicera*, *Amphicercidustuberculatus* David, Narayanan & Rajasingh, 1971 and *Amphicerciduslonicerae* Maity & Chakrabarti, 1982, were found in India and a further two species feeding on *Lonicera*, *Amphicercidussinilonicericola* Zhang, 1980 and *Amphicercidusforsythiae* Zhang, Zhong & Zhang, 1992 were recorded in China. Until now, ten valid species of *Amphicercidus* have been recorded worldwide (Favret 2024).

A systematic revision of the genus in China has not been conducted, and the validity of *A.lonicerae* and *A.sinilonicericola* has been questioned (Blackman and Eastop 2024). Through a systematic investigation of *Amphicercidus* specimens in China, two species are here confirmed as synonyms and two are newly recorded.

## ﻿Materials and methods

Aphid terminology used in this paper follows that of Miyazaki (1971) and Blackman and Eastop (2024), with body length measured from the frons to the end of the cauda. The specimens were examined using a Leica DM 2500 light microscope and photographed with a Leica MC 5400 camera. The unit of measurement is millimeter (mm) in Table 2. The following abbreviations are used in Table 2: Ant. I, II, III, IV, V, and VIb: antennal segments I, II, III, IV, V, and the base of antennal segment VI, respectively; PT: processus terminalis; Ant. III BD: basal diameter of antennal segment III; URS: ultimate rostral segment; BW URS: basal width of ultimate rostral segment; hind tibia MW: mid-width of hind tibia; 2HT: second hind tarsal segment; SIPH: siphunculus; DW SIPH: distal width of siphunculus; BW SIPH: basal width of siphunculus; MW SIPH: mid-width of siphunculus; BW Cauda: basal width of cauda; cephalic setae: the longest seta on vertex; setae on Ant. III: the longest seta on antennal segment III; setae on hind tibia: the longest seta on hind tibia; setae on tergite I: the longest marginal seta on abdominal tergite I; setae on tergite VIII: the longest spinal seta on abdominal tergite VIII.

DNA barcodes of COI were obtained from Chinese specimens and the voucher details are recorded (Table 1). Total genomic DNA was extracted from a single aphid nymph, preserved in 95% or 100% ethanol, using DNeasy Blood & Tissue Kit (Qiagen, Hilden, Germany). The standard DNA barcode gene of aphids was used and amplified with primers LepF and LepR (Foottit et al. 2008). The polymerase chain reaction (PCR) thermal regime was as follows: 5 min initial denaturation at 95 °C followed by 35 cycles of 95 °C for 30s, 52 °C for 30s, 72 °C for 1 min, and a 10 min final extension at 72 °C. The PCR products were sequenced in both directions with BigDye Terminator v. 3.1 Cycle Sequencing Kit (Applied Biosystems, Foster City, CA, USA) and run on an ABI 3730 automated sequencer (Applied Biosystems). Sequences were assembled by SeqMan II (DNASTAR, Inc., Madison, WI, USA) with inspection and manual editing, and then were examined using BLAST to confirm the sequences were highly similar to other aphid sequences. All sequences were deposited in GenBank (Table 1). Multiple alignments were performed with Clustal W (Thompson et al. 1994) and then verified manually. Pairwise genetic distances for the COІ gene were estimated using MEGX (Kumar et al. 2018) under Kimura’s two-parameter (K2P) model (Kimura 1980).

**Table 1.** Voucher and GenBank accession numbers for aphid samples used in the molecular analyses.

Species	Voucher number	Collection locality	Host plant	COI
*Amphicerciduspulverulens*	29606	China: Xizang	*Lonicera*	PQ611218
*Amphicercidustuberculatus*	32720	China: Xizang	*Lonicera*	PQ611219
*Amphicercidustuberculatus*	32721	China: Xizang	*Lonicera*	PQ611220
*Amphicercidusjaponicus*	44055	China: Beijing	*Lonicera*	PQ611221
*Amphicercidusjaponicus*	45445	China: Beijing	*Lonicera*	PQ611222
*Amphicercidusjaponicus*	48119	China: Beijing	*Lonicera*	PQ611223
*Amphicercidusjaponicus*	50282	China: Yunnan	*Lonicera*	PQ611224
*Amphicercidusjaponicus*	030603J01	Korea	*Lonicera*	GU978778*

*Sequences downloaded from GenBank.

The specimens examined are deposited in the National Animal Collection Resource Center, Institute of Zoology, Chinese Academy of Sciences, Beijing, China.

## ﻿Taxonomic results

### ﻿ Amphicercidus

Taxon classificationAnimalia

Oestlund, 1923

2E37C9D0-5830-5412-B3DD-0DD7F4BE1F61

Amphicercidus Oestlund, 1923: 126.

#### Type species.

*Aphispulverulens* Gillette, 1911 by original designation.

#### Diagnosis.

Body elliptical and heavily covered with white wax powder in life; body dorsum smooth, abdominal tergites often with large and round marginal or spinal tubercles; setae of body dorsum and appendages long and pointed; head with undeveloped diverging antennal tubercles; median frontal tubercle flat or slightly swollen; antennal segment III with 3–49 secondary rhinaria in apterae and 2–152 secondary rhinaria in alatae; the second hind tarsal segment at least 1.50× of ultimate rostral segment; siphunculi as long stout cylinder, without flange, smooth or with fine transverse wrinkles; cauda broad, about as long as or slightly longer than its basal width.

The genus resembles *Macchiatiella* Del Guercio, 1909 in antennal segment III with secondary rhinaria and broad cauda, but it can be distinguished by the following characteristics: (1) antennal tubercles undeveloped (in *Macchiatiella*: antennal tubercles well developed); (2) body dorsum without sclerotized markings (in *Macchiatiella*: body dorsum with sclerotized markings); (3) second hind tarsal segment at least 1.50× of ultimate rostral segment (in *Macchiatiella*: second hind tarsal segment shorter than ultimate rostral segment); (4) siphunculi without flange (in *Macchiatiella*: siphunculi with flange). The genus also resembles *Allocotaphis* Börner, 1950 in having broad cauda, but it can be distinguished as follows: (1) abdominal tergites smooth, without sclerotized markings (in *Allocotaphis*: abdominal tergites often with sclerotized markings); (2) setae of body dorsum and appendages long and pointed (in *Allocotaphis*: setae of body dorsum and appendages short and pointed or capitate); (3) antennal segment III with numerous round secondary rhinaria in alatae (in *Allocotaphis*: antennal segment III with protuberant secondary rhinaria); (4) second hind tarsal segment at least 1.50× of ultimate rostral segment (in *Allocotaphis*: second hind tarsal segment shorter than ultimate rostral segment); (5) siphunculi without flange (in *Allocotaphis*: siphunculi with developed flange). The genus also similar to *Anuraphis* Del Guercio, 1907 in having broad cauda, but it can be distinguished as follows: (1) most species feeding on *Lonicera* (Caprifoliaceae) (in *Anuraphis*: the species transfer between *Prunus* (Rosaceae) and Asteraceae or Apiaceae); (2) body dorsum without sclerotized markings (in *Anuraphis*: body dorsum with sclerotized markings) (3) siphunculi long stout cylinder, smooth or with fine transverse wrinkles, without flange (in *Anuraphis*: siphunculi short cylinder and tapering, with close-set rows of minute fine spinules, with distinct flange); (4) second hind tarsal segment at least 1.50× of ultimate rostral segment (in *Anuraphis*: second hind tarsal segment shorter than ultimate rostral segment).

#### Biology.

Most species feed on the shoots and leaves of *Lonicera* (Caprifoliaceae), but *Amphicercidusflocculosus* (Gillette & Palmer, 1929) and *Amphicerciduspulverulens* (Gillette, 1911) feed on *Symphoricarpus* (Caprifoliaceae), and *Amphicerciduslaniger* (Takahashi, 1927) feeds on the young stems of *Stauntonia* (Lardizabalaceae) (Blackman and Eastop 2024).

#### Distribution.

China, Japan, Korea, India, Central Asia, Russia, USA, Canada, Mexico (Blackman and Eastop 2024).

### ﻿ Amphicercidus japonicus

Taxon classificationAnimalia

(Hori, 1927)

F549A924-713E-5B37-9313-969668CE2511

**Figure 1.**
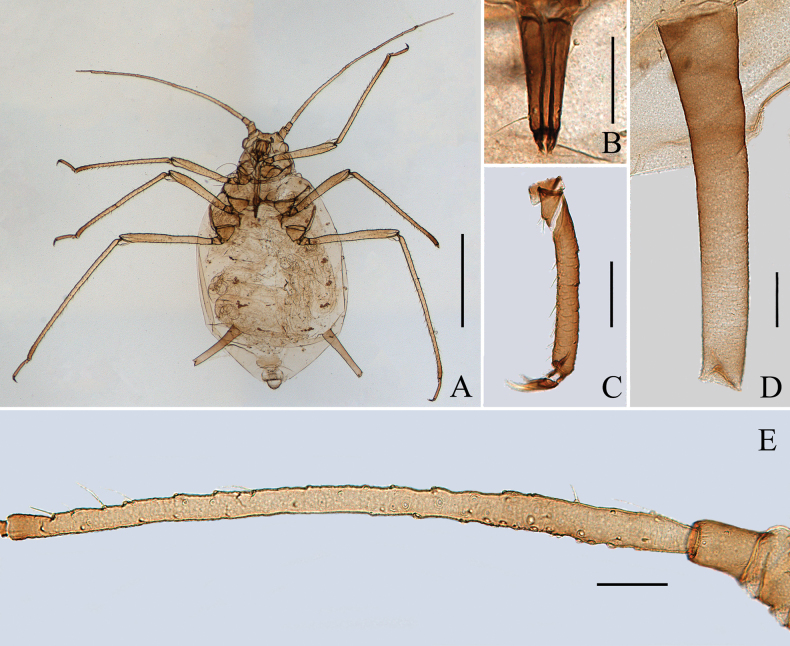
Specimen No. 45445-1-1-2: apterous viviparous female of *Amphicercidusjaponicus* (Hori). **A.** Habitus of body; **B.** Ultimate rostral segment; **C.** Second hind tarsal segment; **D.** Siphunculus; **E.** Antennal segment III. Scale bars: 1.00 mm (**A**); 0.10 mm (**B–E**).

**Figure 2.**
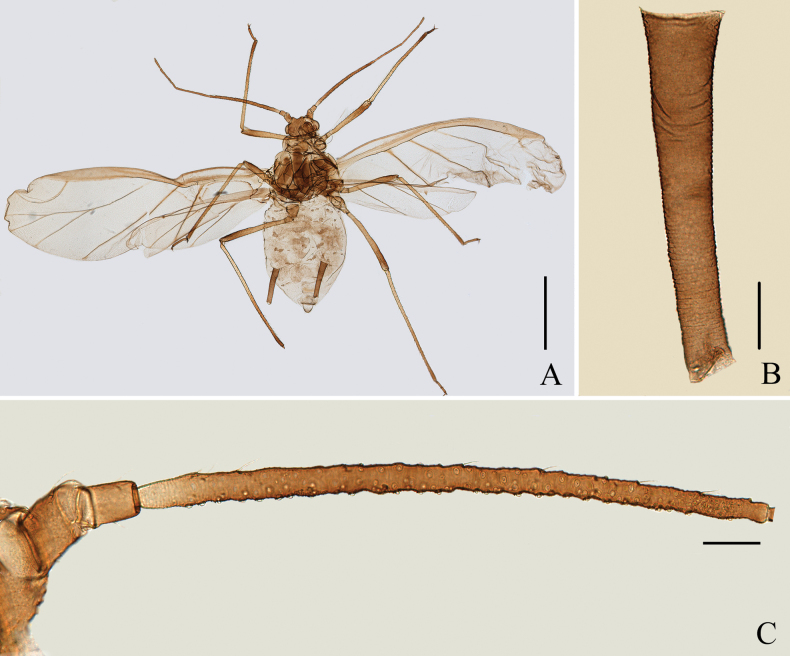
Specimen No. 45445-1-1-1: alate viviparous female of *Amphicercidusjaponicus* (Hori): **A.** Habitus of body; **B.** Siphunculus; **C.** Antennal segment III. Scale bars: 1.00 mm (**A**); 0.10 mm (**B, C**).

**Figure 3.**
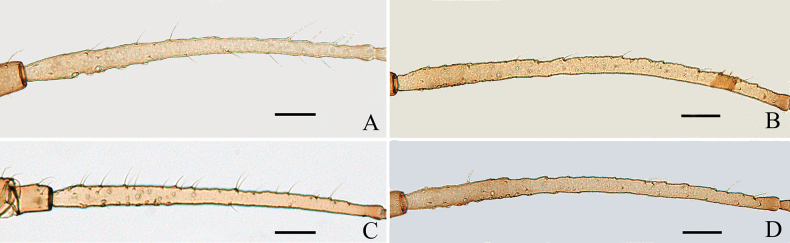
The secondary rhinaria on the antennal segment III of *Amphicercidusjaponicus* (Hori) in apterae shown: **A.** Antennal segment III with 5 secondary rhinaria in specimen No. 51330; **B.** Antennal segment III with 29 secondary rhinaria in specimen No. 48119; **C.** Antennal segment III with 20 secondary rhinaria in specimen No. 44055; **D.** Antennal segment III with 35 secondary rhinaria in specimen No. 45445. Scale bars: 0.10 mm.

**Figure 4.**
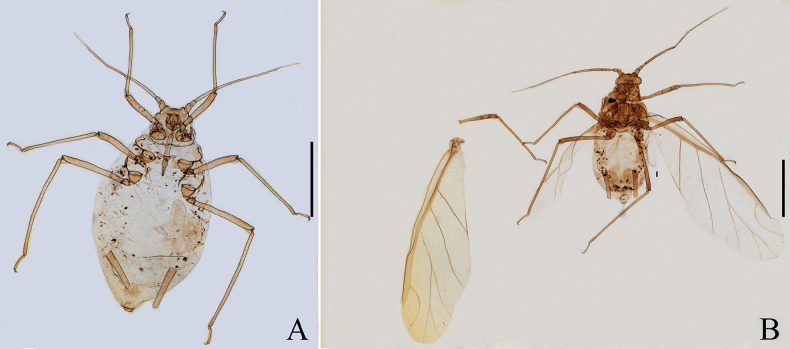
Specimen No. 7150: *Amphicercidusjaponicus* (Hori) (*Amphicercidusforsythiae* Zhang, Zhong & Zhang, 1992 syn. nov.). **A.** Habitus of apterous viviparous female; **B.** Habitus of alate viviparous female. Scale bars: 1.00 mm.

**Figure 5.**
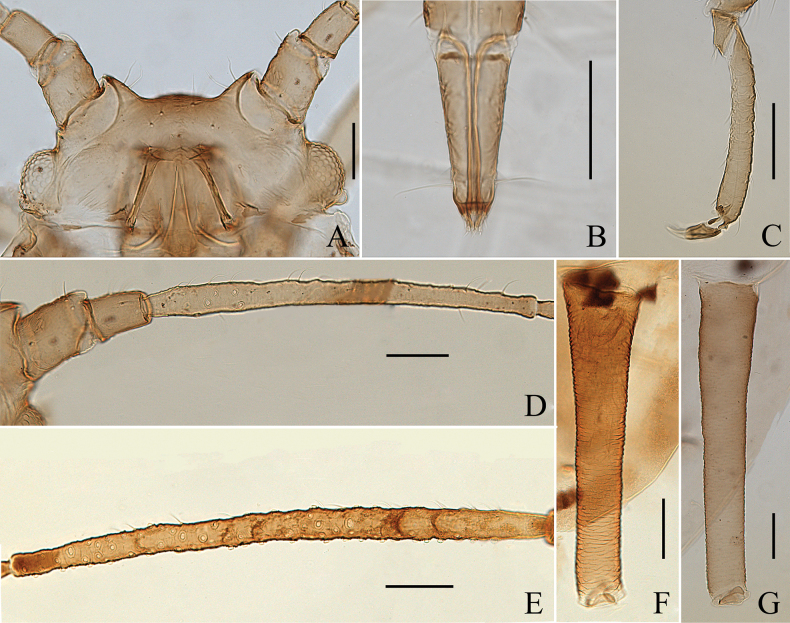
Specimen No. 7150-1-1-2: *Amphicercidusjaponicus* (Hori) (*Amphicercidusforsythiae* Zhang, Zhong & Zhang, 1992 syn. nov.). Apterous viviparous female: **A.** Dorsal view of head; **B.** Ultimate rostral segment; **C.** Second hind tarsal segment; **D.** Antennal segments I-III; **G.** Siphunculus. Alate viviparous female; **E.** Antennal segment III; **F.** Siphunculus. Scale bars: 0.10 mm.

**Figure 6.**
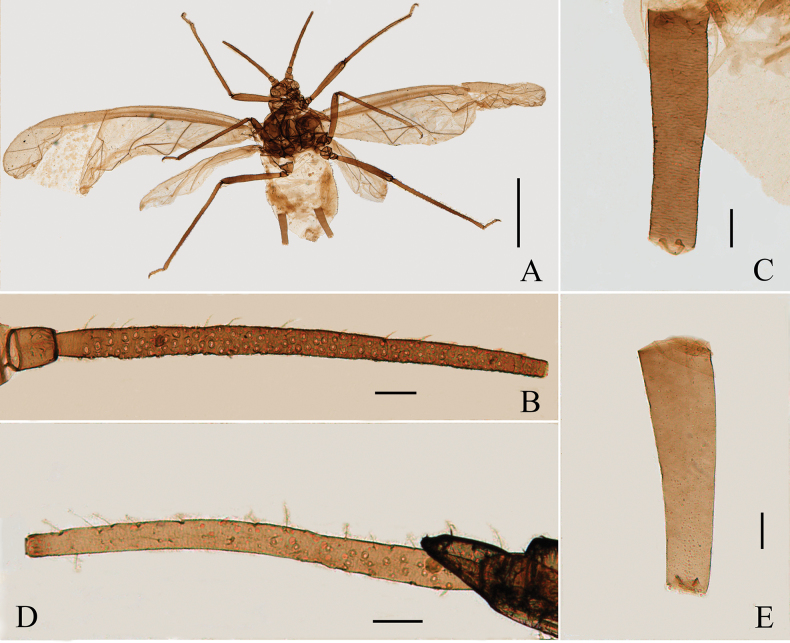
Specimen No. 4108-1-1-1: *Amphicercidusjaponicus* (Hori) (*Amphicercidussinilonicericola* Zhang, 1980 syn. nov.). Alate viviparous female: **A.** Habitus of body; **B.** Antennal segment III; **C.** Siphunculus. Apterous viviparous female; **D.** Antennal segment III; **E.** Siphunculus. Scale bars: 1.00 mm (**A**); 0.10 mm (**B–E**).

Figs 1 , 2 , 3 , 4 , 5 , 6 , 14A–C ; Table 2

Anuraphis japonicus Hori, 1927: 193.
Amphicercidus forsythiae Zhang, Zhong & Zhang, 1992: 196, syn. nov.
Amphicercidus sinilonicericola Zhang, 1980: 53, syn. nov.

#### Types examined.

***Holotype*** and ***paratypes*** of *Amphicercidusforsythiae* Zhang, Zhong & Zhang, 1992: two apterous females and two alate viviparous females, **China: Yunnan** (Lijiang City), 24.V.1980, No. 7150-1-1, on *Lonicera*, coll. T. S. Zhong and L. Y. Wang. ***Holotype*** and ***paratypes*** of *Amphicercidussinilonicericola* Zhang, 1980: two apterous females and three alate viviparous females, **China: Yunnan** (Kunming City), 24.II.1960, No. 4108, on *Lonicera*, coll. Y. F. Han.

**Table 2.** Morphometric data on species of *Amphicercidus* in China based on examined specimens. All measurements are in mm.

Parts		*Amphicercidusjaponicus*		*Amphicercidusjaponicus* (*Amphicercidusforsythiae* Zhang, Zhong & Zhang, 1992), syn. nov.		*Amphicercidusjaponicus* (*Amphicercidussinilonicericola* Zhang, 1980), syn. nov.	*Amphicerciduspulverulens*	*Amphicercidustuberculatus*	
		Apterous viviparous females (*n* = 8)	Alate viviparous females (*n* = 2)	Apterous viviparous females (*n* = 2)	Alate viviparous females (*n* = 2)	Alate viviparous females (*n* = 3)	Alate viviparous females (*n* = 2)	Apterous viviparous females (*n* = 2)	Alate viviparous females (*n* = 2)
Length (mm)	Body length	2.06–3.51	2.92–3.02	2.87–3.23	2.38–2.61	2.66–3.36	2.81–3.01	2.61–3.39	2.81–3.17
	Body width	0.89–1.81	1.24–1.32	1.62–1.80	1.06–1.10	1.23–1.51	1.12–1.14	1.42–1.78	1.24–1.30
	Antennae	1.64–2.74	–	1.89–2.03	2.34–2.57	–	2.51–2.81	2.10–2.81	–
	Ant. I	0.11–0.15	0.15–0.15	0.13–0.13	0.12–0.14	0.12	0.14–0.17	0.13–0.18	0.14–0.15
	Ant. II	0.08–0.11	0.09–0.11	0.09–0.10	0.10	0.12	0.11–0.12	0.11–0.13	0.11
	Ant. III	0.44–1.07	0.98–1.11	0.59–0.65	0.81–0.88	1.18	0.72–0.83	0.57–0.84	0.86
	Ant. IV	0.26–0.54	0.50–0.51	0.31–0.36	0.40–0.43	–	0.44–0.50	0.35–0.50	0.44
	Ant. V	0.26–0.43	0.50–0.51	0.27–0.30	0.33–0.38	–	0.41–0.48	0.31–0.39	0.36
	Ant. VIb	0.14–0.16	0.13–0.13	0.15–0.15	0.16–0.17	–	0.17–0.19	0.15–0.17	0.16
	PT	0.36–0.51	–	0.34–0.35	0.40–0.49	–	0.53–0.54	0.49–0.59	–
	URS	0.15–0.18	0.16–0.17	0.16–0.17	0.14–0.16	0.17–0.18	0.15–0.17	0.15–0.17	0.15–0.16
	Hind femur	0.70–1.24	1.04–1.13	1.00–1.04	0.95–0.96	1.15–1.20	0.99–1.00	0.84–1.15	1.04
	Hind tibia	1.14–1.88	1.85–1.93	1.55–1.65	1.58–1.64	1.94–2.10	1.55–1.66	1.42–1.93	1.82
	2HT	0.23–0.28	0.24–0.27	0.26–0.26	0.25	0.29–0.30	0.25–0.27	0.24–0.28	0.26–0.27
	SIPH	0.43–0.82	0.56–0.62	0.67–0.71	0.54–0.55	0.65–0.67	0.25–0.33	0.64–0.89	0.63
	BW SIPH	0.12–0.16	0.09–0.12	0.14–0.15	0.12–0.13	0.16	0.08–0.09	0.14–0.18	0.10–0.11
	MW SIPH	0.10–0.12	0.08–0.09	0.10	0.08–0.09	0.13	0.07–0.08	0.13–0.14	0.09
	DW SIPH	0.08–0.09	0.08–0.09	0.09–0.10	0.08–0.09	0.11–0.12	0.06–0.07	0.09–0.09	0.09
	Cauda	0.09–0.14	0.13–0.14	0.12	0.09–0.13	0.10–0.13	0.13	0.13–0.16	0.16
	BW Cauda	0.15–0.22	0.18	0.18	0.14–0.16	0.14–0.16	0.17	0.16–0.20	0.18
	Ant. III BD	0.03–0.04	0.04	0.03–0.04	0.03–0.04	0.05–0.06	0.03–0.04	0.03–0.04	0.03
	Hind tibia MW	0.04–0.06	0.04–0.05	0.06	0.04–0.10	–	0.05	0.05–0.06	0.05
	Cephalic setae	0.05–0.07	0.05	0.06	0.05	0.05	0.04	0.08	0.08
	Setae on Tergite I	0.04–0.07	0.04–0.06	0.05	0.03	0.05–0.08	0.04	0.06–0.08	0.07
	Setae on Tergite VIII	0.06–0.08	0.06–0.07	0.06	0.06–0.07	–	0.05	0.09	0.09
	Setae on Ant. III	0.03–0.06	–	0.05	0.04	0.04	0.03	0.06–0.09	0.07
	Setae on Hind tibia	0.06–0.09	0.07	0.08–0.09	0.05–0.07	0.07–0.10	0.07	0.07–0.10	0.07–0.09
Number	Number of secondary rhinaria on Ant. III	3–39	101–103	4–6	90–112	120–123	43–46	3–17	58–59
	Number of secondary rhinaria on Ant. IV	0	0	0	1–4	4	0–8	0	0
	Number of secondary rhinaria on Ant. V	0	0	0	0	0	0–1	0	0
Ratio (times)	Body length / Body width	1.74–2.31	2.28–2.35	1.78–1.80	2.25–2.37	2.16–2.42	2.51–2.63	1.84–1.90	2.27–2.44
	Whole antennae / Body	0.80–0.87	–	0.58–0.71	0.90–1.08	–	0.89–0.93	0.81–0.83	–
	Hind femur / Ant. III	1.18–1.69	0.94–1.15	1.54–1.75	1.08–1.19	0.97–1.01	1.2	1.37–1.48	1.20–1.22
	Hind tibia / Body	0.56–0.59	0.63–0.64	0.51–0.54	0.61–0.69	0.58–0.65	0.55	0.54–0.57	0.57–0.65
	Ant. I / Ant. III	0.15–0.24	0.13–0.15	0.21–0.22	0.14–0.17	0.10	0.19–0.20	0.21–0.23	0.16–0.17
	Ant. II / Ant. III	0.11–0.19	0.08–0.11	0.15–0.16	0.11–0.13	0.10	0.14	0.16–0.19	0.13
	Ant. IV / Ant. III	0.45–0.61	0.45–0.52	0.52–0.56	0.49–0.50	–	0.60	0.59–0.63	0.51
	Ant. V / Ant. III	0.42–0.59	0.34–0.40	0.45–0.46	0.41–0.44	–	0.57	0.47–0.55	0.42
	Ant. VIb / Ant. III	0.17–0.33	0.14	0.24–0.26	0.19–0.20	–	0.23	0.20–0.27	0.18
	PT / Ant. III	0.53–0.81	–	0.54–0.58	0.49–0.56	–	0.65	0.70–0.86	–
	PT / Ant. VIb	2.29–3.28	–	2.25–2.26	2.44–2.90	–	2.87	3.21–3.43	–
	URS / BW URS	2.50–2.68	2.45–3.00	2.23–2.69	2.30–2.59	1.57–1.66	2.34–2.62	2.22–2.38	2.03–2.19
	URS / 2HT	0.61–0.66	0.62–0.68	0.62–0.64	0.55–0.63	0.57–0.62	0.58–0.62	0.61–0.63	0.57–0.60
	Cauda / BW Cauda	0.58–0.69	0.73–0.77	0.67	0.63–0.80	0.68–0.83	0.78	0.78–0.80	0.86–0.89
	Cephalic setae / Ant. III BD	1.36–2.14	1.28	1.59–1.97	1.37	0.90	1.22	2.11–2.70	2.52–2.55
	Setae on Tergite I / Ant. III BD	1.25–2.10	1.13–1.57	1.30–1.30	0.94	0.92–1.45	1.16	2.03–2.18	2.12–2.15
	Setae on Tergite VIII / Ant. III BD	2.05–2.62	1.44–2.03	1.57	1.88–1.94	–	1.13	2.32–2.90	2.73–2.82
	Setae on ANT. III / ANT. III BD	0.96–1.76	–	1.38–1.45	1.00–1.03	0.74–0.77	0.80	2.07–2.29	2.09–2.12
	Setae on hind tibia / Hind tibia MW	1.11–1.70	1.48–1.55	1.32–1.52	0.52–1.66	0.96–1.44	1.50	1.17–2.00	1.58–2.00
	SIPH / Body	0.21–0.24	0.19–0.20	0.22–0.23	0.21–0.23	0.19–0.21	0.09–0.11	0.25–0.26	0.20–0.22
	SIPH / Cauda	5.22–6.21	4.03–4.78	–	4.30–6.30	5.17–6.21	1.97	5.13–5.45	3.97–3.99
	SIPH / Ant. III	0.73–0.98	0.51–0.63	1.04–1.20	0.62–0.68	0.54–0.57	0.35–0.39	1.06–1.13	0.73
	SIPH / BW SIPH	3.49–6.23	4.59–6.71	4.49–4.94	4.18–4.59	3.70–4.28	3.02–3.48	4.45–5.08	5.85–6.04
	SIPH / MW SIPH	4.44–7.91	6.78–7.23	6.61–7.12	5.83–6.85	4.35–5.29	3.79–4.19	4.58–7.11	7.14–7.20
	SIPH / DW SIPH	5.20–10.04	6.56–7.43	6.88–8.38	5.96–6.85	4.98–5.55	4.62–5.03	7.37–9.99	6.75–7.03

Parts *Amphicercidusjaponicus* (*Amphicercidusforsythiae* Zhang, Zhong & Zhang, 1992), syn. nov. *Amphicercidusjaponicus* (*Amphicercidussinilonicericola* Zhang, 1980), syn. nov. *Amphicerciduspulverulens*, *Amphicercidustuberculatus*

#### Additional material examined.

Other specimens of *Amphicercidusjaponicus* (Hori, 1927): two apterous females, • **China: Beijing** (Baihua Mountain; alt. 1077 m), 3.VII.2018, No. 44055-1-1, on *Lonicera*, coll. H. Long; one apterous female (COI: PQ611221), • **China: Beijing** (Baihua Mountain), 3.VII.2018, No. 44055-1-1, on *Lonicera*, coll. H. Long; two apterous females, • **China: Beijing** (Baihua Mountain; alt. 731 m), 19.V.2019, No. 45445-1-1, on *Lonicera*, coll. H. Long; one apterous females (PQ611222), • **China: Beijing** (Baihua Mountain), 19.V.2019, No. 45445-1-1, on *Lonicera*, coll. H. Long; one alate viviparous female, • **China: Beijing** (Baihua Mountain: alt. 731 m), 19.V.2019, No. 45446-1-1, on *Lonicera*, coll. H. Long; one apterous female, • **China: Yunnan** (Lijiang City; alt. 2416.78 m), 26.V.2021, No. 50282-1-1. on *Lonicera*, coll. T. Y. Liu and S. Xu; one apterous female (COI: PQ611224), • **China: Yunnan** (Lijiang City), 26.V.2021, No. 50282-1-1. on *Lonicera*, coll. T. Y. Liu and S. Xu; one alate viviparous female and two apterous females, • **China: Beijing** (alt. 43.67 m), 11.V.2021, No. 48119-1-1, on *Lonicera*, coll. Y. Xu; one apterous female (COI: PQ611223), • **China: Beijing**, 11.V.2021, No. 48119-1-1, on *Lonicera*, coll. Y. Xu; one apterous female, • **China: Xizang** (Motuo county; 1757.5 m), 25.VI.2022, No. 51330-1-1, on *Lonicera*, coll. Z. X. Li; one nymph, • **China: Sichuan** (Aba City; alt. 3259.5 m); 22.VI.2021, No. 51598-1-1, on *Lonicera*, coll. T. Y. Liu and S. Xu.

#### Diagnosis.

Dorsal tubercles small and round, irregularly distributed on abdominal tergites II–V; antennal segment III with 3–39 secondary rhinaria in apterae and with more than 90 secondary rhinaria in alatae; processus terminalis 1.60–2.80× as long as the base of this segment (Figs 1–3).

#### Comment.

The validity of *Amphicercidusforsythiae* Zhang, Zhong & Zhang, 1992 and *A.sinilonicericola* Zhang, 1980 has been in doubt (Blackman and Eastop 2024). In the original descriptions, the primary differences between *A.japonicus*, *A.forsythiae*, and *A.sinilonicericola* are the number of secondary sensoria (Fig. 3), the length of setae on antennal segment III, the number of accessory hairs on the ultimate rostral segment, and the ratio of siphunculi length to head width across the eyes (Zhang et Zhong 1980; Zhang et al. 1992). However, after examining approximately twenty specimens collected in China, the number of accessory hairs on the ultimate rostral segment are 6–8, siphunculi are longer than head width, and other characteristics are overlapped. Therefore, the evidence is insufficient to support the validity of *Amphicercidusforsythiae* and *A.sinilonicericola*.

*Amphicercidusforsythiae* (Figs 4, 5) was distinguished from *A.japonicus* by the mesosternal furca with a long stem, antennal segment III with 4–6 circular secondary rhinaria in apterae and the longest setae on antennal segment III 0.79× of the basal width (Zhang et al. 1992). After examining the holotype and paratypes of *A.forsythiae*, these features are also present in *A.japonicus* and its morphological characteristics are extremely similar to those of *A.japonicus*. The only differences are following: *A.forsythiae* has slightly protuberant antennal tubercles (Fig. 5A) and siphunculi 1.04–1.20× of antennal segment III, while *A.japonicus* has undeveloped antennal tubercles and siphunculi 0.73–0.98× of antennal segment III. However, when the specimens are well-prepared, *A.japonicus* also has slightly protuberant antennal tubercles (Fig. 1A). The quantitative characteristics are a not sufficient to support the validity of *A.forsythiae*. Therefore, *Amphicercidusforsythiae* Zhang, Zhong & Zhang, 1992 is regarded as the junior synonym of *Amphicercidusjaponicus* (Hori, 1927).

*Amphicercidussinilonicericola* (Fig. 6) was distinguished from *A.japonicus* by dorsal seta of head 0.93× basal width of antennal segment III, antennal segment III with 113–163 circular secondary rhinaria in alate, ultimate rostral segment with four secondary setae, mesosternal furca with a long stem, and siphunculi longer than head width across the eyes (Zhang et Zhong 1980; Blackman and Eastop 2024). After examining the holotype and paratypes of *A.sinilonicericola* Zhang, 1980 (Fig. 6), its morphological characteristics are extremely similar to those of *A.japonicus*, with differences only in the following characteristics: ultimate rostral segment wide wedge-shaped, 1.57–1.66× its basal width in alatae (in *A.japonicus*: ultimate rostral segment wedge-shaped, 2.45–3.00× its basal width in alatae); antennal segment III with 120–123 secondary rhinaria in alatae (Fig. 6B) (in *A.japonicus*: antennal segment III with 101–103 secondary rhinaria (Fig. 2C) in alatae). Considering that these quantitative characters can be affected by the specimen preparation process, it is impossible to accurately classify them as two species based on these characters. So, *Amphicercidussinilonicericola* Zhang, 1980 is regarded as a junior synonym of *Amphicercidusjaponicus* (Hori, 1927).

#### Biology.

The species feed on the shoots and leaves of *Lonicera* (Caprifoliaceae) (Hori 1927; Zhang and Zhong 1980; Zhang et al. 1992; Blackman and Eastop 2024).

#### Distribution.

China (Beijing, Liaoning, Shaanxi, Sichuan, Xizang, Yunnan); Japan; Korea; India; Russia.

### ﻿ Amphicercidus laniger

Taxon classificationAnimalia

(Takahashi, 1927)

32B234CE-F833-5BE0-9881-D20C3381D0ED

Anuraphis lanigera Takahashi, 1927: 7.

#### Diagnosis.

Abdominal tergites lacking distinct tubercles; antennal segment III without secondary rhinaria in apterae and with 2–5 secondary rhinaria in alatae; processus terminalis 2.20× of the basal part of last antennal segment; feed on *Stauntonia* (Lardizabalaceae) (Takahashi 1927).

#### Biology.

The species feeds on the young stems of *Stauntonia* (Lardizabalaceae) (Takahashi 1927).

#### Distribution.

China (Taiwan).

##### ﻿New records for China

### ﻿ Amphicercidus pulverulens

Taxon classificationAnimalia

(Gillette, 1911)

ABB8DA85-E26B-536A-A2D6-9C50564459EF

**Figure 7.**
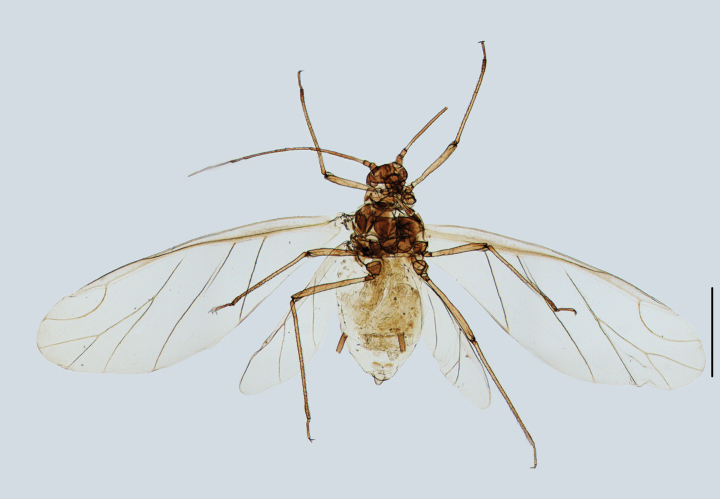
Specimen No. 25978-1-1: *Amphicerciduspulverulens* (Gillette): habitus of alate viviparous female. Scale bar: 1.00 mm.

**Figure 8.**
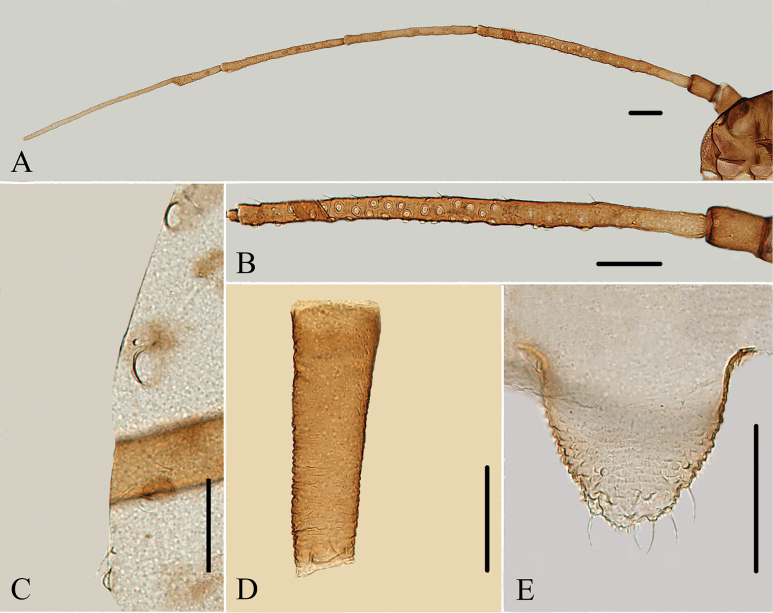
Specimen No. 25978-1-1: *Amphicerciduspulverulens* (Gillette). Alate viviparous female: **A.** Antenna; **B.** Antennal segment III **C.** Marginal tubercles on abdominal tergites II-IV; **D.** Siphunculus; **E.** Cauda. Scale bars: 0.10 mm.

**Figure 9.**
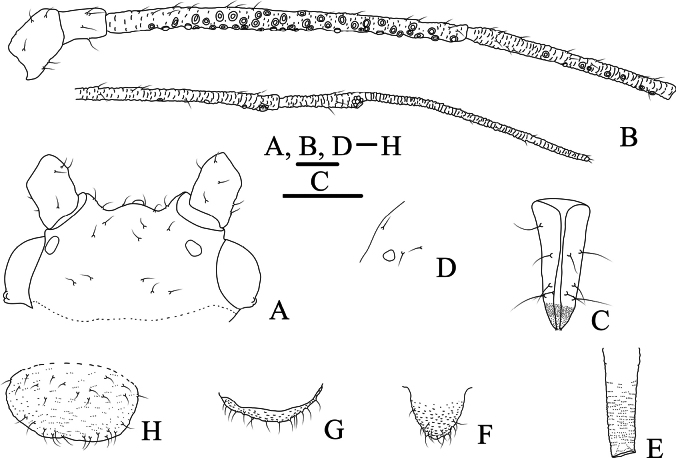
Specimen No. 25978: *Amphicerciduspulverulens* (Gillette). Alate viviparous female: **A.** Dorsal view of head; **B.** Antenna; **C.** Ultimate rostral segment; **D.** Marginal tubercle on abdominal tergite II; **E.** Siphunculus; **F.** Cauda; **G.** Anal plate; **H.** Genital plate. Scale bars: 0.10 mm.

Figs 7 , 8 , 9 , 14D , Table 2

Aphis pulverulens Gillette, 1911: 324.

#### Specimens examined.

One alate viviparous female, **China: Xizang** (Linzhi City; alt. 2000 m), 4.VIII.2014, No. 29606-1-1, on *Lonicera*, coll. J. Chen and X. C. Zhu; one alate viviparous female (COI: PQ611218), **China: Xizang** (Linzhi City), 4.VIII.2014, No. 29606-1-1, on *Lonicera*, coll. J. Chen and X. C. Zhu; one alate viviparous female, **China: Xizang** (Linzhi City; alt. 3900 m), 5.VIII.2014, No. 29578-1-1, on *Lonicera*, coll. J. Chen and X. C. Zhu.

#### Diagnosis.

Pronotum and abdominal tergites I-V each with one pair of marginal tubercles (Fig. 8C); siphunculi short stout cylinder, as long as second hind tarsal segment (Figs 8D, 9E), 0.09–0.11× body length; antennal segment III with 43–46 secondary rhinaria in alatae (Figs 8B, 9B); processus terminalis 2.87–3.14× the base of the last antennal segment.

#### Comment.

The species has the shortest siphunculi in the genus and occurs on *Symphoricarpos* (Caprifoliaceae) in USA and Canada. After checking the syntype USNMENT00399393.001 of *A.pulverulens* from the USA (Smithsonian National Museum of Natural History, 2025), it is a new record for Chinese aphid fauna. This is the first record of *A.pulverulens* on *Lonicera* (Caprifoliaceae) in China. The species population found in China is very similar to the original description, with siphunculi being short and stout cylinder, as long as second hind tarsal segment, without flange; the Ant. III as long as Ant. III+IV; cauda short and broad. But, the characteristics of this species exhibit some differences between those found in China and the original records. In China, the species is orange in life, with straight siphunculi, whereas in USA, it is dusty green in life, and the siphunculi are weakly curved (Gillette 1911; Oestlund 1923).

#### Biology.

The species feeds on the young stems of *Symphoricarpos* (Caprifoliaceae) in USA and Canada (Gillette 1911; Blackman and Eastop 2024), but this is the first record of it feeding on *Lonicera* (Caprifoliaceae) in China.

#### Distribution.

China (Xizang); USA; Canada.

### ﻿ Amphicercidus tuberculatus

Taxon classificationAnimalia

David, Narayanan & Rajasingh, 1971

9DC130B2-44E4-5F14-B0CC-877BECB3429D

**Figure 10.**
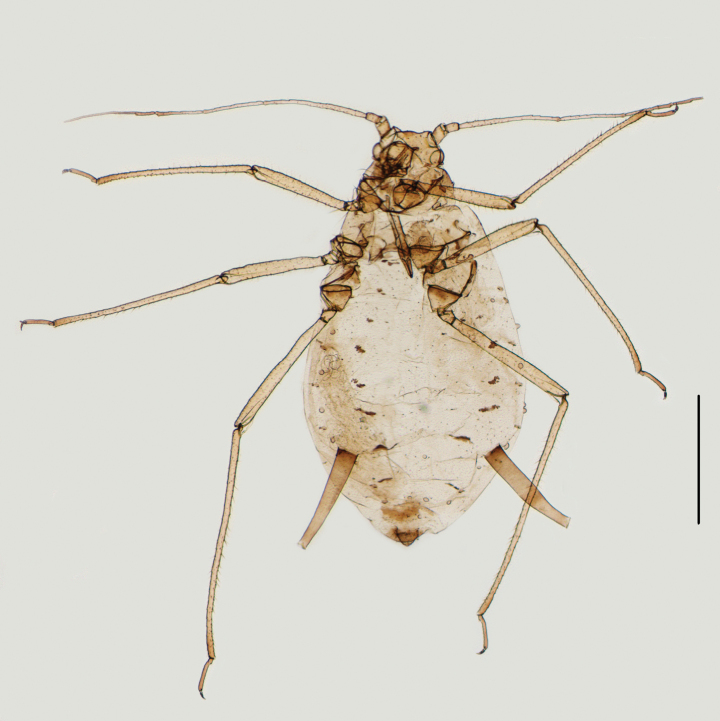
Specimen No. 32721-1-1-2: *Amphicercidustuberculatus* David, Narayanan & Rajasingh: habitus of apterous viviparous female. Scale bar = 1.00 mm.

**Figure 11.**
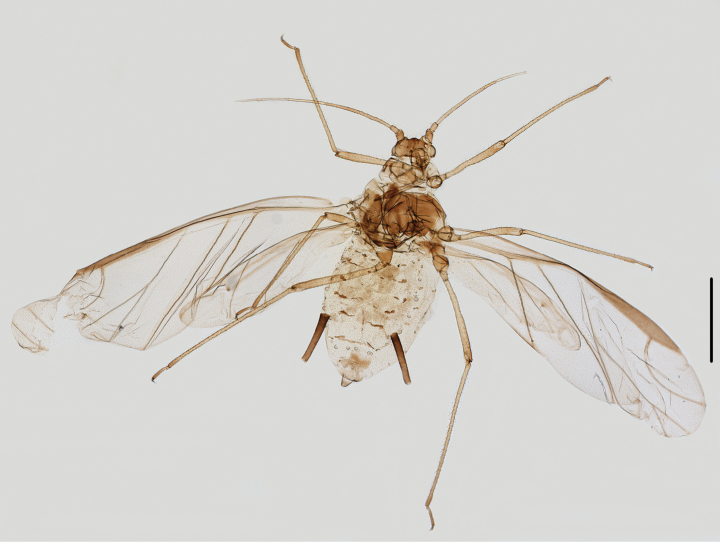
Specimen No. 32721-1-1-1: *Amphicercidustuberculatus* David, Narayanan & Rajasingh: habitus of alate viviparous female. Scale bar = 1.00 mm.

**Figure 12.**
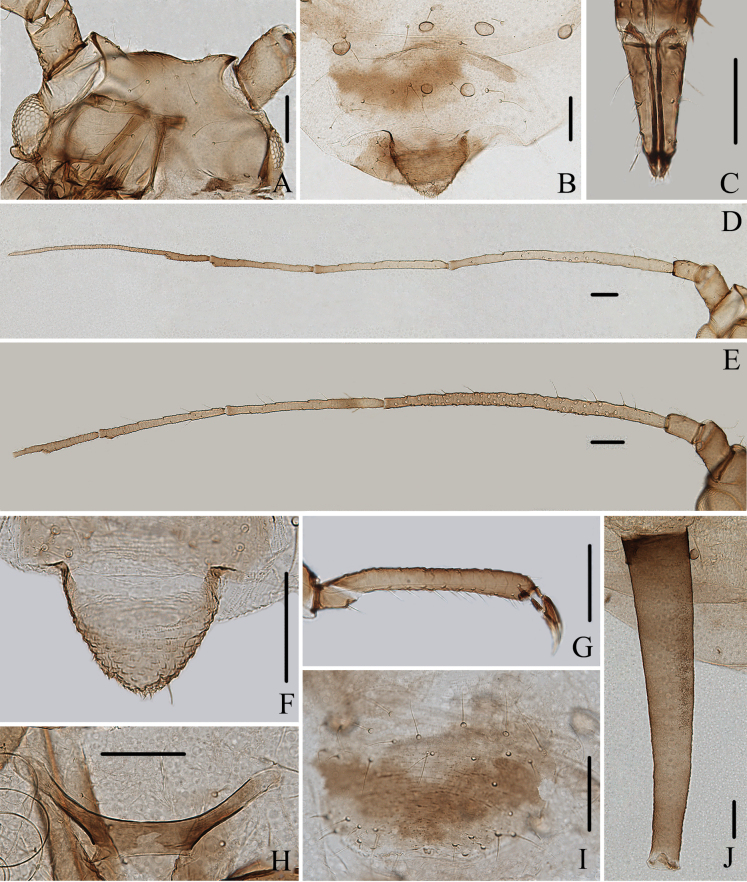
Specimen No. 32721-1-1-2: *Amphicercidustuberculatus* David, Narayanan & Rajasingh. Apterous viviparous female: **A.** Dorsal view of head; **B.** Spinal tubercles of abdominal tergites VII-VIII **C.** Ultimate rostral segment; **D.** Antenna; **F.** Cauda; **G.** Second hind tarsal segment; **H.** Mesosternal furca siphunculus; **I.** Genital plate; **J.** Siphunculus. Alate viviparous female; **E.** Antennal segments I-VIb. Scale bars: 0.10 mm.

**Figure 13.**
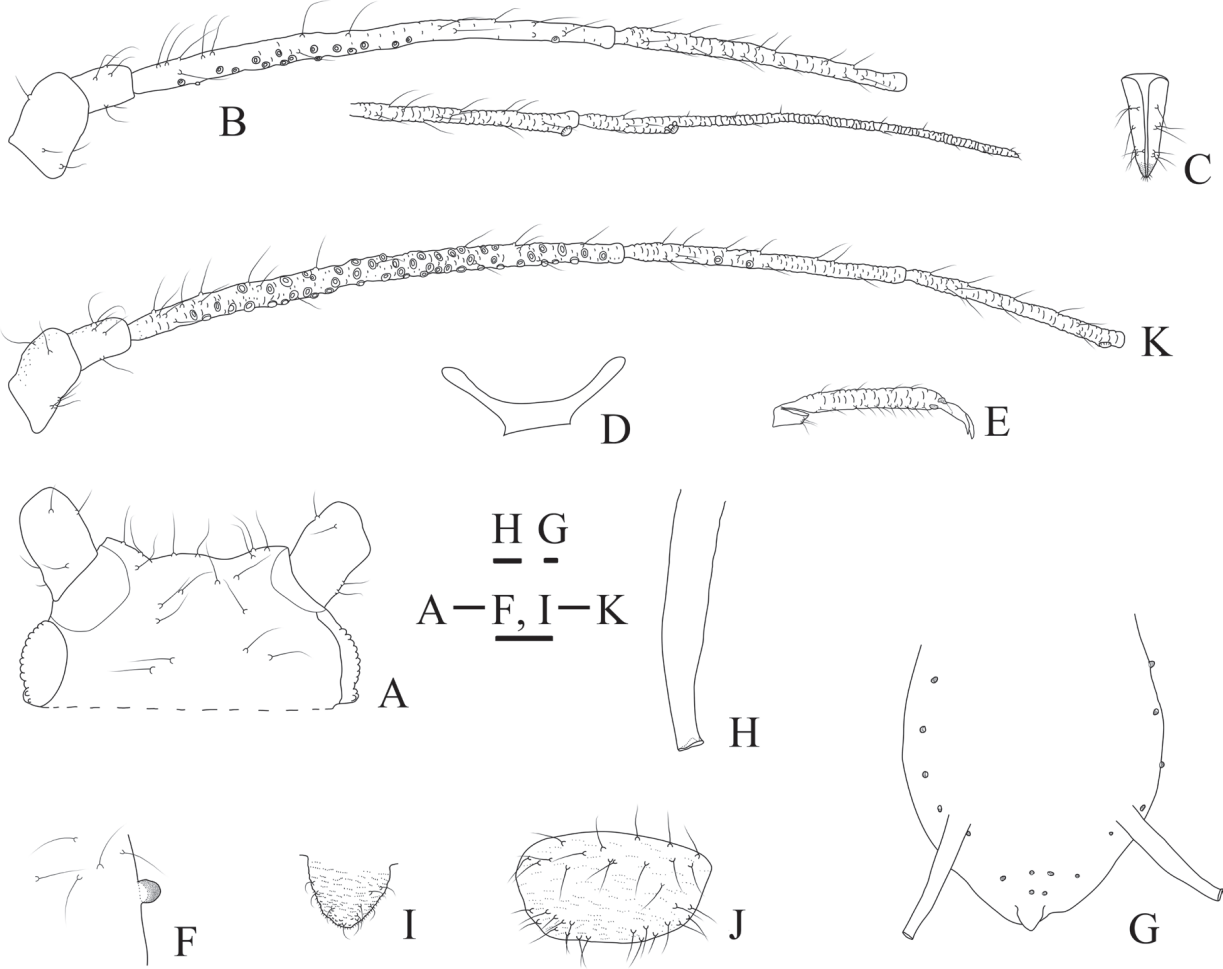
Specimen No. 32721-1-1-2: *Amphicercidustuberculatus* David, Narayanan & Rajasingh. Apterous viviparous female: **A.** Dorsal view of head; **B.** Antenna; **C.** Ultimate rostral segment; **D.** Mesosternal furca; **E.** Second hind tarsal segment; **F.** Marginal tubercle of abdominal tergite I; **G.** Marginal and spinal tubercles on abdomen; **H.** Siphunculus; **I.** Cauda; **J.** Genital plate. Alate viviparous female **K.** Antenna segments I-V. Scale bars: 0.10 mm.

**Figure 14.**
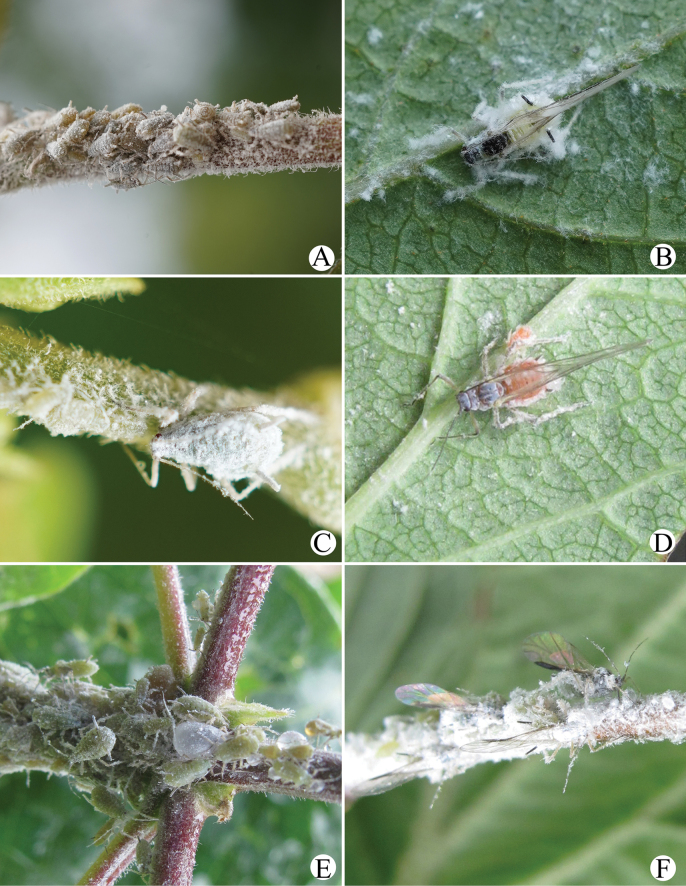
*Amphicercidus* species in the field. **A.** Apterae of *A.japonicus* feeding on tender stem of *Lonicera*; **B.** Alatae of *A.japonicus* feeding on the underside of leaves of *Lonicera*; **C.** Apterae of *A.japonicus* feeding on tender stem of *Lonicera*; **D.** Alatae of *A.pulverulens* feeding on the underside of leaves of *Lonicera*; **E.** The apterae of *A.tuberculatus* feeding on tender stem of *Lonicera*; **F.** Apterae and alatae of *A.tuberculatus* feeding on tender stem of *Lonicera*.

Figs 10 , 11 , 12 , 13 , 14E, F , Table 2

Amphicercidus tuberculatus David, Narayanan & Rajasingh, 1971: 419.

#### Specimens examined.

One alate viviparous female and one apterous female, 23.VII.2014, **China: Xizang** (Shigatse City; alt. 2657 m), No. 32720-1-1, on *Lonicera*, coll. J. Chen and X. C. Zhu; one apterous female (COI: PQ611219), 23.VII.2014, **China: Xizang** (Shigatse City), No. 32720-1-1, on *Lonicera*, coll. J. Chen and X. C. Zhu; one alate viviparous female and one apterous female, 23.VII.2014, **China: Xizang** (Shigatse City; alt. 2657 m), No. 32721-1-1, on *Lonicera*, coll. J. Chen and X. C. Zhu; one apterous female (COI: PQ611220), 23.VII.2014, **China: Xizang** (Shigatse City), No. 32721-1-1, on *Lonicera*, coll. J. Chen and X. C. Zhu.

#### Diagnosis.

Dorsal tubercles on body are large and round; pronotum and abdominal tergites II–VII each with one pair of marginal tubercles, and abdominal tergites VII and VIII each with one pair of spinal tubercles (Figs 12B, 13G); antennal segment III with 3–17 secondary rhinaria in apterae (Figs 12D, 13B); processus terminalis 3.21–3.43× as long as the basal part of the last antennal segment.

#### Comment.

The species resembles *Amphicercidusjaponicus* (Hori), but the latter may be distinguished by having the following characters: body dorsum with small tubercles, irregularly distributed on the abdomen; antennal segment III with 16–39 secondary rhinaria in apterae; and the processus terminalis 2.41–3.11× as long as the basal part of the segment.

#### Biology.

The species feeds on feeds on the young stems and leaves of *Lonicera* (Caprifoliaceae) (David et al. 1971; Blackman and Eastop 2024).

#### Distribution.

China (Xizang); India.

##### ﻿DNA barcoding

The DNA barcodes of the three species of *Amphicercidus* (*A.japonicus*, *A.pulverulens*, and *A.tuberculatus*) from China were obtained, and the barcodes of *A.pulverulens* and *A.tuberculatus* were acquired for the first time. The final alignment sequences of COI consisted of 658 nucleotides, including 24 parsimony-informative sites. The interspecific genetic distances between *A.japonicus* and *A.pulverulens* is 2.80–3.00%, between *A.japonicus* and *A.tuberculatus* is 3.75–4.08%, and between *A.pulverulens* and *A.tuberculatus* is 4.56–4.57%. Based on DNA barcodes, the validity of these species is supported.

### ﻿Key to species of *Amphicercidus* in China

**Table:** 

1	Abdominal tergites lacking distinct tubercles; antennal segment III without secondary rhinaria in apterae and with 2–5 secondary rhinaria in alatae; on *Stauntonia*	***A.laniger***
–	Abdominal tergites with distinct rounded tubercles; antennal segment III with at least 6 secondary rhinaria in apterae and alatae; on Caprifoliaceae	**2**
2	Siphunculi as short cylinder, 0.09–0.11× body length, 2.00× cauda	***A.pulverulens***
–	Siphunculi as long and thick cylinder, at least 0.20× body length, at least 4.00× cauda	**3**
3	Abdominal tergites II-VI each with one pair of developed and round marginal tubercles and tergites VII-VIII each with one pair of spinal tubercles; processus terminalis 2.90–3.70× as long as the basal part of the last antennal segment; antennal segment III with 6–11 secondary rhinaria in apterae; on *Lonicera*	***A.tuberculatus***
–	Abdominal tergites II-VIII with irregularly arranged round tubercles; processus terminalis 1.60–2.80× as long as the basal part of the last antennal segment; antennal segment III with 3–39 secondary rhinaria in apterae; on *Lonicera*	***A.japonicus***

### ﻿Updated key couplets to aphids feeding on Lonicera in Blackman and Eastop, 2024

**Table:** 

53	(Fund.) Dorsal and antennal hairs short and inconspicuous. SIPH 0.08–0.09× BL, and > 5× longer than their basal widths. ANT without sec. rhinaria. Marginal and spinal tubercles (MTu and STu) not evident. (All progeny of fund. are alate)	***Loniceraphisparadoxa*** *
–	Dorsal and antennal hairs long and fine-pointed. SIPH 0.09–0.26× BL, thick, 3–6× longer than their basal width. ANT with sec. rhinaria. MTu and STu often present	**54**
54	SIPH long and thick cylinder, at least 2× longer than 2HT, at least 0.20× body length, at least 4.00× cauda	***A.pulverulens***
–	SIPH short cylinder, as long as 2HT, 0.09–0.11× of body length, 2.00× cauda	**55**
55	ANT III with only 3–4 rhinaria (except in alatiform individuals). Cauda with 6 hairs	**56**
–	ANT III with 6–49 rhinaria. Cauda with 6–19 hairs	**57**

## ﻿Discussion

The broad and rounded cauda is a generic distinctive morphological character in Macrosiphini, encompassing around thirty genera in the world (Shaposhnikov 1950). According to incomplete statistics, there are 12 genera with broad and rounded cauda belonging to the tribe Macrosiphini in China, which can be identified using the key below.

### ﻿Key to Chinese Macrosiphini genera with broad cauda having a length shorter than its basal width

**Table:** 

1	Siphunculi pore-shaped or short conic; ultimate rostral segment acute, stiletto-shaped	***Cryptosiphum***
–	Siphunculi cylindrical; ultimate rostral segment wedge-shape	**2**
2	Cauda with semicircular basal part and pointed finger-shaped distal part, and the hind margin of anal plate indented to two conical tubercles in apterae	***Acerocaudaphis***
–	Cauda semicircular, helmet- or triangular-shaped, and anal plate semicircular in apterae	**3**
3	Antennal segment III with secondary rhinaria in apterae	**4**
Antennal segment III without secondary rhinaria in apterae	**6**
4	Abdominal tergites without sclerotized markings; the second hind tarsal segment at least 1.50× ultimate rostral segment; siphunculi as stout cylinder, without flange	***Amphicercidus***
–	Abdominal tergites with sclerotized markings; the second hind tarsal segment shorter than ultimate rostral segment; siphunculi as long cylinder, with flange	**5**
5	Antennal segments III and IV with secondary rhinaria in alatae; cauda semicircular	***Macchiatiella***
–	Antennal segments III-V with secondary rhinaria in alatae; cauda escutcheon-shaped	***Allocotaphis***
6	Dorsal setae thick and long; dorsum covered with densely papillate tubercles; siphunculi as long cylinder, without flange	***Tenuilongiaphis***
–	Dorsal setae short and pointed, or long and pointed; dorsum smooth or with small spicules; siphunculi as short cylinder, with flange	**7**
7	Siphunculi swollen at middle part, shorter than triangular cauda	***Brevicoryne***
–	Siphunculi cylindrical, not swollen, longer than cauda	**8**
8	Abdominal tergites I-V with paired and large rounded marginal and spinal tubercles; siphunculi cylinder	***Dysaphis***
–	Dorsum without marginal tubercles or only with small marginal tubercles; siphunculi truncate conical or thick cylinder	**9**
9	Siphunculi truncate conical or cylindrical, with a subapical annular incision	***Brachycaudus***
–	Siphunculi cylindrical, without subapical annular incision	**10**
10	Ultimate rostral segment long and thin, wedge-shaped, > 2.00× of the second hind tarsal segment	***Oedisiphum***
–	Ultimate rostral segment wedge-shaped, less than 1.50× the second hind tarsal segment	**11**
11	Siphunculi long cylindrical, with long and pointed setae; first tarsal chaetotaxy: 4, 4, 2; abdominal tergites with a large central sclerite in alatae	***Sorbaphis***
–	Siphunculi short cylindrical, without setae; first tarsal chaetotaxy: 3, 3, 3; abdominal tergites with transverse bands in alatae	***Sappaphis***

## Supplementary Material

XML Treatment for Amphicercidus

XML Treatment for Amphicercidus japonicus

XML Treatment for Amphicercidus laniger

XML Treatment for Amphicercidus pulverulens

XML Treatment for Amphicercidus tuberculatus

## ﻿Additional information

### Author ORCIDs

Ying Xu https://orcid.org/0000-0002-8950-7718

Jing Chen https://orcid.org/0000-0002-7584-5249

Li-Yun Jiang https://orcid.org/0000-0002-2527-9613

Ge-Xia Qiao https://orcid.org/0000-0002-7300-6812

### Data availability

All of the data that support the findings of this study are available in the main text.
